# Factors associated with poor knowledge among adults on tuberculosis in Bangladesh: results from a nationwide survey

**DOI:** 10.1186/s41043-015-0002-4

**Published:** 2015-05-01

**Authors:** Shahed Hossain, Khalequ Zaman, Abdul Quaiyum, Sayera Banu, Ashaque Husain, Akramul Islam, Martien Borgdorff, Frank van Leth

**Affiliations:** 1 Centre for Equity and Health Systems (CEHS), International Centre for Diarrhoeal Disease Research, Bangladesh (icddr,b), Mohakhali, Dhaka, 1212 Bangladesh; 2 International Centre for Diarrhoeal Disease Research, Bangladesh (icddr,b), Mohakhali, Dhaka, 1212 Bangladesh; 3 National TB Control Programme (NTP), DGHS, Dhaka, Bangladesh; 4 Health Programme, BRAC, Dhaka, Bangladesh; 5 Department of Clinical Epidemiology, Academic Medical Centre, University of Amsterdam, Amsterdam, The Netherlands; 6 Centre for Infection and Immunity Amsterdam, Academic Medical Centre, University of Amsterdam, Amsterdam, The Netherlands; 7 Public Health Service, Amsterdam, The Netherlands; 8 Department of Global health, Academic Medical Centre, Amsterdam Institute for Global Health and Development, University of Amsterdam, Amsterdam, The Netherlands; 9 KNCV Tuberculosis Foundation, The Hague, The Netherlands

**Keywords:** Tuberculosis, Knowledge, Community, Bangladesh

## Abstract

**Introduction:**

In 2012, Bangladesh continues to be one of the 22 high tuberculosis (TB) burden countries in the world. Although free diagnosis and management for TB is available throughout the country, case notification rate/100,000 population for new smear positive (NSP) cases under the national TB control programme (NTP) remained at around 70/100,000 population and have not changed much since 2006. Knowledge on TB disease, treatment and its management could be an important predictor for utilization of TB services and influence case detection under the NTP. Our objective is to describe knowledge of TB among newly diagnosed TB cases and community controls to assess factors associated with poor knowledge in order to identify programmatic implications for control measures.

**Methods:**

Embedded in TB prevalence survey 2007–2009, we included 240 TB cases from the TB registers and 240 persons ≥ 15 years of age randomly selected from the households where the survey was implemented. All participants were interviewed using a structured, pre-tested questionnaire to evaluate their TB knowledge. Regression analyses were done to assess associations with poor knowledge of TB.

**Results:**

Our survey documented that overall there was fair knowledge in all domains investigated. However, based on the number of correct answers to the questionnaires, community controls showed significantly poorer knowledge than the TB cases in the domains of TB transmission (80% vs. 88%), mode of transmission (67% vs. 82%), knowing ≥ 1 suggestive symptoms including cough (78% vs. 89%), curability of TB (90% vs. 98%) and availability of free treatment (75% vs. 95%). Community controls were more likely to have poor knowledge of TB issues compared to the TB cases even after controlling for other factors such as education and occupation in a multivariate model (OR 3.46, 95% CI: 2.00-6.09).

**Conclusions:**

Knowledge on various aspects of TB and TB services varies significantly between TB cases and community controls in Bangladesh. The overall higher levels of knowledge in TB cases could identify them as peer educators in ongoing communication approaches to improve care seeking behavior of the TB suspects in the community and hence case detection.

## Background

Bangladesh started the implementation of Directly Observed Treatment, Short Course (DOTS), the World Health Organization’s (WHO) advocated strategy for tuberculosis (TB) control, in 1993. Under the DOTS strategy, diagnosis and treatment of TB was made available free of cost from over 1000 centres throughout the country. However, after 20 years of DOTS implementation, Bangladesh has remained one of the 22 high TB burden countries in the world, with an estimated prevalence of new smear positive (NSP) TB cases among adults above ≥ 15 years as 79.4/100,000 [1–3]. The case notification rate for NSP TB in Bangladesh remained static from 2006 onwards at around 70/100,000 per year [2]. It has also been observed that in over 50% of cases care seeking for TB-related symptoms starts in the informal sector and often remains outside of the National TB control Programme (NTP) for several health care visits, potentially leading to treatment delay and ongoing transmission [4].

DOTS is a therapeutic based approach which largely depends on the willingness of the affected person to accept and adhere to it. This is particularly true in Bangladesh, where case detection under the NTP is passive. Care seeking decisions are often based on knowledge of a disease, availability of services and the perceived quality of care. Knowledge therefore could be an important predictor of not only of initial care seeking behavior but also indirectly of the course of the disease and its outcomes [5–8].

Considering the importance of people’s knowledge of the availability and effectiveness of the DOTS programme, the NTP and its partners have been engaged in Advocacy Communication and Social Mobilization (ACSM) campaigns, largely funded by the Global Fund to fight AIDS, TB and Malaria (GFTAM). These campaigns should raise the general population’s awareness of the importance of TB, the need for early diagnosis, and the availability of free TB treatment at DOTS facilities. The impact of these ACSM campaigns has never been fully measured. A recent TB-monitoring report for the country identified considerable shortcomings in the organization of ACSM activities that question their potential efficacy [9]. These shortcomings include inadequate human resources to carry out the activities, inadequate attention to urban settings, and failure to lay out a clear plan to address stigma, discrimination and gender issues [9].

There is no direct evidence on how well ACSM campaigns can convey information, but anecdotal evidence suggests that the information provided may not reach the intended population in a meaningful way [10]. We therefore added a sub-study to the latest national TB prevalence survey, which was carried out in 2007–2009. Our objective was to assess knowledge of TB symptoms, routes of transmission, and treatment options. Furthermore we explored factors associated with poor knowledge. Apart from assessing the overall level of knowledge of TB in the country, we assessed whether recently diagnosed TB cases differed in their knowledge compared to the community controls, and could therefore potentially serve as “peer educators”. It is expected that information from this study might have implications for future planning of TB-control measures in Bangladesh.

## Methods

Setting and sampling: This study was a part of the national TB prevalence survey which was carried out in 2007–2009 [3]. This was a population based cross-sectional survey, which randomly included 40 clusters, equally from urban and rural areas, using a proportion to population size sampling strategy. The sub-districts in rural areas and zones in urban areas were primary sampling units (PSU). From each unit, one *mauza or muhallh* (lowest level administrative unit) was selected as a study cluster. Households were selected in each cluster from a random starting point with consecutive inclusion until the estimated target size of 1400 individuals per cluster was reached. A total of 52089 individuals (≥15 years of age) were included in the national survey [3].

For this study, we included the six most recently registered pulmonary TB (PTB) cases from the local DOTS centre and six adults randomly selected from the survey’s sampling frame as a comparison group from each cluster. This provided a sample of 240 TB cases and 240 adults from the general community population. All included cases were new smear positive (NSP) pulmonary TB cases, passively diagnosed through the ongoing NTP programme. All cases were on treatment at time of inclusion in the study. Community controls were selected contemporary to the TB cases from each cluster. No refusal was reported in this survey. When an individual was absent a second attempt was made to reach her/him. Only one individual per household was included.

Data Collection: Information on TB knowledge was obtained through face-to-face interviews with the participants by trained interviewers. The interviewers were trained one week at Dhaka and further one week at field level in a pilot cluster. A structured questionnaire was prepared based on NTP monitoring guidelines [11], and other knowledge studies [12–16]. The questionnaire included socio-demographic and TB knowledge items. The questionnaire was translated into Bangla and pretested for its clarity and understandability before finalization. Pretesting of the questionnaire was done in Dhaka and in a cluster area not included in the sample. Pretesting was performed by the supervisors and the results were discussed among the research team for necessary modification before finalization. There were two field level supervisors and one lab supervisor in each of the team engaged in the survey. The supervisors were qualified researchers with at least 10 years of field level experiences in conducting surveys. All questionnaires were checked by the supervisor for accuracy and completeness. The supervisor also re-interviewed 5% of all cases to cross check the accuracy of information. Every alternate cluster was included for cross checking equally from rural and urban areas. No major discrepancies were reported.

Measurements: The interview collected information on demographic parameters including age, sex, and area of residence, levels of education, and occupation status. The questions on TB knowledge included the domains of symptoms, transmission and treatment.

Statistical approach: Data were checked, processed and analyzed by using the Statistical package STATA 12.0 (Stata Corp. TX. U.S.A.). Residence was dichotomized in urban and rural setting. Age and occupation were categorized into three groups, and education into four based on obtained frequencies. Occupation was divided into three categories: ‘manual labour’ (including agriculture workers, day labourer, rickshaw pullers etc.), ‘sales and services’ (including shop keepers, office workers, administrators etc.), and ‘dependants’ (including housewives, students, unemployed persons). All TB-knowledge questions were categorized into correct or incorrect according to NTP guidelines [11]. We compared the proportions of participants providing correct answers for each knowledge item among TB cases and community controls. We generated a ‘knowledge score’ as the composite score of five key questions (1 for correct, 0 for incorrect answer) resulting in a range of 0 to 5. The key questions explored the common communication messages of the national and global TB programme ACSM activities, which included that (i) TB is contagious, (ii) TB is transmitted by airborne spread, (iii) cough is a main symptom of TB, (iv) TB is curable and (v) TB treatment is available free at DOTS centre. The NTP has used these messages for about two decades based on their high sensitivity (chronic cough as a symptom of TB) and specific programme related information [11–16]. From these messages we derived the five questions and developed the variable “TB-knowledge” assigning ‘poor’ to those who answered only 0–3 correctly, and ‘fair’ to those answered 4 or 5 correctly. Univariable regression analyses assessed the association between the demographic variables and “poor TB-knowledge”. In the multivariable analyses, the association of interest was between TB-knowledge and type of participant (TB cases vs community control). We included only those dependent variables that changed the odds ratio of the initial association by more than 10%. We assumed that TB-knowledge is directly linked with levels of education, and tested for effect modification between each of the variables and education, by means of the log-likelihood ratio test.

Ethical consideration: Ethical clearance was obtained from the institutional review board (IRB, FWA-00001468) of the International Centre for Diarrhoeal Diseases Research, Bangladesh (icddr,b). Written consent was obtained from all participants.

## Results

Approximately half of the study population was between 15–34 years of age in both groups with significantly more males among TB cases (63%) than community controls (48%). There were also significant differences between the TB cases and community controls in their distribution of education, occupation and smoking status (Table 1).

**Table 1:** **Socio demographic characteristics of the participants**

**Characteristics**	**TB cases 240, n (%)**	**Community control 240, n (%)**	P value*
**Age groups (Years)**			
15-34	112 (46.7)	127 (52.9)	0.150
35-54	75 (31.3)	76 (31.7)	
≥55	53 (22.0)	37 (15.4)	
**Sex**			
Male	152 (63.3)	116 (48.3)	0.001
Female	88 (36.7)	124 (51.7)	
**Residence**			
Rural	120 (50.0)	120 (50.0)	1.000
Urban	120 (50.0)	120 (50.0)	
**Education**			
0	101 (42.1)	78 (32.5)	0.001
1-5	65 (27.1)	47 (19.6)	
6-10	62 (25.8)	85 (35.4)	
10+	12 (5.0)	30 (12.5)	
**Occupation**			
“Sales & services”	109 (45.4)	33 (13.8)	0.001
“Manual labour”	108 (53.7)	93 (38.7)	
“Dependents”	23(9.6)	114 (47.5)	
**Smoker**			
Yes	128 (64.0)	72 (36.0)	0.001
No	112 (40.0)	168 (60.0)	

*p < 0.05, Chi X^2^ values.

TB cases in comparison to community controls provided significantly more correct answers for most TB-knowledge questions (Table 2). That TB is a contagious disease was correctly identified by 88.8% and 80.4%, and TB transmission modes by 82.5% and 67.1%, by the TB cases and community controls respectively. There was no difference between the two groups in identifying cough as a TB symptom. However, other TB-related symptoms were more often correctly identified by TB cases compared to community controls. Correct answers to the questions related to programmatic aspects of TB management were high in both groups, but significantly higher among TB cases. As a result, TB cases had a higher overall ‘TB-knowledge score’ than community controls (Table 2).

**Table 2:** **Knowledge about TB among adult TB cases and community controls**

**Knowledge items**	**Includes correct answer**		
	**TB cases n (%)**	**Community control n (%)**	p-value*
**TB transmission**			
TB contagious/Infectious	213 (88.8)	193 (80.4)	0.011
TB transmits by air (Cough/sneeze)	198 (82.5)	161 (67.1)	0.001
**Symptoms of PTB**			
Know ≥ 1 suggestive symptoms including cough	210 (87.5)	188 (78.3)	0.001
Cough	215 (89.5)	207 (86.5)	0.263
Fever	188 (78.3)	144 (60.0)	0.000
Chest pain	158 (65.8)	131 (54.6)	0.012
Loss of weight	187 (77.9)	127 (52.9)	0.001
Blood in cough	204 (85.0)	114 (47.0)	0.001
Difficult breathing	153 (63.8)	127 (52.9)	0.016
Night sweating	112 (46.7)	51 (21.5)	0.001
Loss of appetite	178 (74.2)	111 (46.2)	0.001
**TB treatment, programmatic information**			
TB curable	237 (98.8)	216 (90.0)	0.001
TB treatment free	229 (95.4)	180 (75.0)	0.001
Knowledge score (Median IQR)^?^	5 (4–5)	4 (3–5)	0.001

*p < 0.05 Chi X^2^ values,^?^ Ranksum test.

Table 3 shows the results of the regression analyses. The univariable logistic regression showed significantly more often “poor knowledge” in community controls compared to TB-cases (OR: 3.12, 95% CI: 1.96-4.97; p = 0.001). The odds of “poor knowledge” was higher in women compared to men (OR: 2.04, 95%CI 1.32-3.16), urban compared to rural residents (OR: 1.75, 95%CI: 1.12-2.71), and “dependents” compared to other occupational groups (OR: 2.22. 95%CI: 1.28-3.84). Having had formal education was associated with a lower odds of “poor knowledge” (Table 3).

**Table 3:** **Factors related to poor knowledge on TB**

**Factors**	**TB Knowledge**	**Univariable**		**Multivariable**	
	**Poor n (%)**	**OR (95% CI)**	**p-value**	**OR (95% CI)**	**p-value**
**Case status**					
TB case^a^	31 (12.9)	1		1	
Community control	76 (31.7)	3.12 (1.96-4.97)	0.001	3.46 (2.00-6.09)	0.001
**Sex**					
Male^d^	45 (16.8)	1			
Female	62 (29.2)	2.04 (1.32-3.16)	0.001		
**Age groups in years**					
15-34^c^	59(24.7)	1			
35-54	27 (17.9)	0.66 (0.39-1.10)	0.116		
≥55	21 (23.3)	0.92 (0.52-1.64)	0.799		
**Education in Years**					
None^b^	53 (29.6)	1		1	
1 – 5	17 (15.8)	0.43 (0.23-0.78)	0.026	0.43 (0.23-0.81)	0.008
6 or more	37 (19.6)	0.59 (0.36-0.94)	0.006	0.47 (0.27-0.80)	0.006
**Residence**					
Rural^e^	42 (17.5)	1			
Urban	65 (27.1)	1.75(1.12-2.71)			
**Occupation**					
Sales & Services^f^	27 (19.0)	1		1	
Manual labour	33 (16.4)	0.83 (0.47-1.46)	0.533	0.60 (0.33-1.09)	0.092
Dependents	47 (34.3)	2.22 (1.28-3.84)	0.004	0.95 (0.49-1.84)	0.873

Note: From a multiple logistic model including a) case status, TB cases as reference category, b) education modeled as dummy variables with the category ‘0 or no education’ as reference group, c) agegroup modeled as dummy variables with category 15–34 years as reference group), d)sex, male as reference group, e) residence, rural as reference group f)occupation modeled as dummy variables with the category ‘sales & service’ as reference group.

Among the demographic variable examined, only education and occupation changed the odds ratio between TB cases or community controls and TB-knowledge, and were therefore included in the multivariable model as potential confounders. In this model, community controls were still significantly more likely to have poor knowledge compared to TB cases (OR 3.46, 95% CI: 2.00-6.09) (Table 3). We did not find any significant effect modification by education.

## Discussion and conclusions

The present study reported an overall fair level of knowledge of cough as an important symptom of TB, ways of transmission, curability and availability of free TB treatment in Bangladesh. Despite this, knowledge of community controls of TB symptoms other than cough and the availability of free treatment was markedly lower than that of TB cases. Overall, community controls had more than 3 times higher odds of having “poor knowledge” compared to TB cases.

A recent study among TB patients at urban DOTS centres in Bangladesh also examined knowledge of TB symptoms [17]. Only 61% of the participants recognized “cough more than 3 weeks” as an important symptom of TB and only 56% knew that TB could be transmitted through “sneezing and coughing”. More than 90% of the respondents reported that TB could be cured and that drugs are given through DOTS centre [17]. Data from 2007 Demographic Health Survey (DHS) from country wide sample of ever married women (~11,000) also showed an adequate level of knowledge that TB is curable (78%), but poor knowledge on transmission (7%) [18]. Similar studies of TB knowledge in other countirtres reported vairable rates of community knowledge of TB symptoms, transmissions and curability [13, 14, 19–21]. None of the studies linked knowledge in the general population directly to ACSM activities, making the value of these activities difficult to judge.

The significant association of community controls who had never had TB with “poor knowledge” in comparison to TB cases in this study was probably due to the recent exposure of TB cases to information during their diagnosis and treatment under DOTS, which usually includes a counseling session. The significant association of persons without education with poor TB-knowledge implies that information about TB may not be reaching this group in an effective manner. We do not know of TB ACSM activities in Bangladesh that clearly differentiated their mode of delivery for specific groups such as those without formal schooling. Tasnim *et al.* reported the main source of information was from television (46.8%), followed by physicians (18.2%) and family members or friends (14.6%) [17]. The role of print media (3.3%) and non-governmental organization (NGO) workers (7.9%) was less than other sources. However, the scenario could be different in rural areas in terms of context, language and modes of delivery of messages and all of these should be considered [17]. The efficacy of modes of information delivery was explored in a recent study in Pakistan [22]. The researchers found that awareness of TB was higher among urban dwellers and literate people among those who were exposed to mixed such as both media and community members. The study also found that people from different backgrounds might have different preferences for source of information.

The association of “poor knowledge” with urban residency may be related to the larger presence of NGOs in rural compared to urban areas in Bangladesh. The NTP DOTS programme also started at least 10 years later in the urban setting compared to the rural setting. The relatively worse conditions in urban areas of Bangladesh is also seen in other important public health issues, like low immunization coverage among urban slum children and the high proportion of malnutrition among this population group [23]. Dissemination of information by individuals with TB in closely knit communities in the rural areas may have also contributed to the higher level of TB-knowledge in those communities.

In our study, women were more likely than men to have poor TB-knowledge (Table 3). Perhaps, this could explain the persistent lower case detection in women compared to men in Bangladesh [24]. However, it remains unclear if higher levels of TB-knowledge themselves actually lead to improved care seeking behaviour, diagnosis and treatment. A recent systematic review identified gender-specific differences in TB stigma including financial and social barriers that may account for lower case detection rates among women compared to men [25].

The higher association of the ‘dependents’ than other occupation groups with poor TB knowledge in this study probably can be explained by that the ‘dependents’ included a large number of housewives, beggars and unemployment persons. These groups of people also are more likely to have less education, less access to information and have less exposure to social interactions.

Although the NTP of Bangladesh has put a strong emphasis on ACSM activities, up to now there has been no formal evaluation of the impact of these activities. The current study provides some indirect evidence that ACSM activities might have an effect in increasing awareness of TB in the general population. Key elements of TB like the importance of cough, the transmissibility and operational characteristics were known by the majority of people. Areas where TB knowledge can be improved were also identified, including additional symptoms other than cough, and operational issues like the availability of free treatment.

Our study suggests that TB cases could be a useful source to provide information about TB. Not only is their knowledge of disease adequate, they will have a personal experience and may be better able to address societal aspects of the disease specific to their communities. A patient friendly atmosphere is important for the NTP to provide services that woman and illiterate or poor individuals will not shy away from [24]. Peers educators can play an important role in this regard and have been shown to be an effective intervention in HIV-prevention strategies among the youth [26]. In India, Van Rompay *et al.* described a peer-led programme aimed at increasing HIV-awareness in rural communities with high rates of illiteracy. Educators were women’s self-help group leaders and barbers. The authors concluded that the programme was an effective way of disseminating culturally appropriate health-related messages [27].

In the field of TB, peer educators have been used in preventive strategies in universities, and hard-to-reach populations like the homeless and injecting drug users [28, 29]. Such strategies resulted in higher levels of understanding of the disease, and the need for early diagnosis and treatment. Particularly, in settings where most health care seeking starts in the informal sector [30, 31]. Although it is unlikely that the level of knowledge is the sole driver of care seeking behaviour for TB, addressing knowledge gaps in the population could prove beneficial for TB-control in general. The role of a DOTS club comprising TB patients, local support groups, local religious group or work place peers could be explored. Similar experiences reported from, Chile, Peru, Ethiopia and Vietnam demonstrated that peer support groups like a TB club led to significant improvements in access, utilization and outcomes [32].

Our study provides insight into levels of TB-knowledge of the general population in Bangladesh. However we need more information on care seeking behaviours. A recent nation-wide study in Bangladesh examined care seeking behaviour among TB cases [4]. Over 50% of the cases started care seeking with informal providers not related to the NTP. Moreover, nearly half of them remained in the informal sector at multiple subsequent points of care seeking [4]. The main reason delays in seeking care was the perception that symptoms were not serious enough. The role of the severity of disease in care seeking was also previously reported by Karim *et al*. in a large qualitative study in Bangladesh [24]. They emphasized the involvement of TB cases in interactive communication and education activities to convey the importance of early care seeking for TB-related symptoms [24].

Our study represents a nationwide sample of TB patients and community controls and hence provides representative information on TB- knowledge. The main limitation of the study is that it was conducted in those sites where the prevalence survey was carried out, potentially “sensitizing” the study population for TB. If anything, this effect would be expected to have reduced the differences between the TB cases and community controls.

The study provides useful information on TB knowledge among the community people and TB cases. This information could be helpful for ongoing ACSM activities to rethink and reorganize its strategies and targets. With the availability and abundance of mobile phones and other digital media, innovative and more individualized approaches can also be tried to reach those populations in need. For example, NTP or its partners could explore the effect of cross media synergy on access to TB information by different sects of people and its ultimate impact on case detection, reducing delays and enhancing treatment adherence. It is also important to explore the possible role of treated TB patients as peer educators in these activities.
